# Intracranial Arteriovenous Malformations During Pregnancy and Puerperium—A Retrospective Nationwide Population-Based Cohort Study

**DOI:** 10.1227/neu.0000000000003067

**Published:** 2024-06-27

**Authors:** Anni Pohjola, Teresa Vest, Liisa Verho, Karoliina Aarnio, Kirsi Rantanen, Hannele Laivuori, Mika Gissler, Aki Laakso, Mika Niemelä, Petra Ijäs

**Affiliations:** * Department of Neurosurgery, Helsinki University Hospital, Helsinki, Finland;; ‡ Department of Neurology, University of Helsinki and Helsinki University Hospital, Helsinki, Finland;; § Obstetrics and Gynecology, University of Helsinki and Helsinki University Hospital, Helsinki, Finland;; ‖ Medical and Clinical Genetics, University of Helsinki and Helsinki University Hospital, Helsinki, Finland;; ¶ Institute for Molecular Medicine Finland, Helsinki Institute of Life Science, University of Helsinki, Helsinki, Finland;; # Department of Obstetrics and Gynecology, Faculty of Medicine and Health Technology, Center for Child, Adolescent, and Maternal Health Research Tampere, Tampere University Hospital and Tampere University, Tampere, Finland;; ** Department of Knowledge Brokers, Finnish Institute for Health and Welfare, Helsinki, Finland;; †† Department of Molecular Medicine and Surgery, Karolinska Institutet, Stockholm, Sweden;; ‡‡ Region Stockholm, Academic Primary Health Care Centre, Stockholm, Sweden

**Keywords:** Arteriovenous malformation, AVM, Delivery, Pregnancy, Puerperium

## Abstract

**BACKGROUND AND OBJECTIVES:**

The knowledge about the management of patients with brain arteriovenous malformations (AVM) during pregnancy is limited, owing partly to insufficient evidence about the outcomes of newborns. This study aims to explore symptomatic AVMs and their outcomes during pregnancy, delivery, and the postpartum period.

**METHODS:**

We conducted a retrospective analysis by combining patients with symptomatic AVM from a nationwide population-based cohort of all women with a pregnancy resulting in delivery during 1987 to 2016 (n = 1 773 728 deliveries) and our AVM database (n = 805, 1942-2014). Cerebrovascular events during pregnancy were identified through International Classification of Diseases-9, International Classification of Diseases-10, or surgical procedure codes from the Hospital Discharge and Medical Birth Registers. Our analysis focused on treatment characteristics and outcomes of patients with AVM hemorrhage or symptomatic AVM during pregnancy, delivery, or puerperium.

**RESULTS:**

A total of 28 women with symptomatic AVMs during pregnancy, delivery, or postpartum period were followed for an average of 12.8 years (SD = 15.5) after admission. Among them, 21 (75%) experienced AVM hemorrhages during pregnancy, puerperium, or delivery. The mean age of patients was 28.9 years (SD = 5.5). Hemorrhages occurred predominantly during the second (n = 9, 43% of all ruptures) or the third trimester (n = 5, 24%). Two AVM ruptures occurred during labor. Treatment for AVM took place during pregnancy (n = 7, 25%) or puerperium (n = 3, 14%) in 10 patients (35.7%). Only 5 mothers (17.8%) had not been previously pregnant. There was no significant difference in mean Apgar scores between those with AVM hemorrhage (8.3) and those without (8.4).

**CONCLUSION:**

Most mothers in the study had prior pregnancies, suggesting a potentially weaker association between AVM rupture and pregnancy compared to previous reports. Notably, 2 AVM ruptures occurred during spontaneous vaginal deliveries. Outcomes were generally favorable in both mothers and infants. More research is needed to refine our understanding of the optimal timing for invasive treatment during pregnancy.

**ABBREVIATION:**
ICD: International Classification of Diseases
SIPP-FIN: Stroke in Pregnancy and Puerperium in Finland.

Approximately 5% to 12% of maternal deaths during pregnancy and puerperium are caused by hemorrhagic stroke, with maternal mortality rates ranging from 7% even up to 83%.^1,2^ Intracerebral hemorrhage (ICH) during pregnancy can be caused by pre-eclampsia, cortical venous thrombosis, ruptured intracranial aneurysm, or arteriovenous malformation (AVM).^2^ Previous studies have associated pregnancy with increased number of hemorrhagic strokes from cerebrovascular lesions.^3,4^ Recently published study about pregnancy-related ICH reported that AVM was the cause of hemorrhage in 18% of the mothers.^5^ However, the role of pregnancy or delivery in the rupture risk of AVMs remains unclear.^6-9^ As a recent meta-analysis concluded, data on the treatment or outcomes after ruptured AVM during pregnancy remain vague.^10^ Because all modern patient cohorts are highly selected, creating generalizable management protocols for pregnancy-related AVMs is complicated. Because of this, high-quality descriptive cohort studies are required.

We performed a retrospective population-based cohort study of all women with a stroke during pregnancy between the years 1987 and 2016 in Finland (n = 1 773 728 deliveries).^11^ From them and from our pre-existing retrospective AVM database of 805 patients from 1942 to 2016, we selected all women with hemorrhage due to AVM or epileptic seizures caused by AVM during pregnancy, delivery, or puerperium. They were included in this observational study of 28 mothers and their newborns, which aims to describe both maternal and fetal outcomes in the rare event of symptomatic AVM during pregnancy or puerperium.

## METHODS

### Stroke in Pregnancy and Puerperium in Finland Cohort

We retrospectively collected a nationwide population-based cohort of all women with pregnancy resulting in delivery during 1987 to 2016 (1 773 728 deliveries). Hospital Discharge Register and Medical Birth Register were used to identify women with cerebrovascular events during pregnancy using International Classification of Diseases (ICD)-9, ICD-10, or surgical procedure codes. Detailed description about the formation of Stroke in Pregnancy and Puerperium in Finland (SIPP-FIN) cohort has been reported previously.^12^ Register data were validated and stroke type and temporal connection to pregnancy verified from patient records obtained from healthcare facilities across Finland. From the maternal strokes identified, patients with ICH (n = 48) or subarachnoid hemorrhage (n = 55) were further analyzed for vascular malformations. Out-of-hospital deaths were identified using the Register of Causes of Death based on ICD codes 6740A and O99.4. Death certificates were analyzed to determine mortality related to AVM.

### Helsinki AVM Cohort

We expanded our data search by using our pre-existing AVM database (n = 805) collected during 1942 to 2014. We further identified 7 patients, who had experienced AVM rupture during pregnancy, delivery, or puerperium. The earliest case found was from 1956. The inclusion of the older patient series allowed us to illustrate the outcomes in the more conservative era of AVM management. The Helsinki AVM database was collected retrospectively through medical records and images. The database includes all patients with AVM treated in the Helsinki district, which serves approximately 40% of the Finnish inhabitants and is responsible for providing care for demanding and rare diseases for patients from all over Finland. The AVM diagnosis, the level of extirpation after invasive treatment, possible associated aneurysms, AVM size, and venous drainage pattern were analyzed using angiography (computed tomography and/or digital subtraction angiography) and/or MRI. Before 1978, AVM diagnosis was mostly based on carotid angiography and the hemorrhage status on blood in cerebrospinal fluid sample. Lesions were classified in Spetzler-Martin grades, which were converted into Spetzler-Ponce.^13^ Follow-up data were obtained from the medical records and partly from a health-related quality of life (HRQOL) questionnaire. The HRQOL questionnaires were sent in 2016 to all living adult patients (n = 432) in the database.

### Case Identification

We reviewed all clinical records and radiological findings of patients in the final study cohort to verify AVM diagnosis and identify potential duplicates. We included only patients with ruptured or symptomatic AVMs during pregnancy, delivery, or puerperium to focus on cases where clinical presentation could affect treatment decisions or pregnancy outcomes. While our data may not include all patients with undiagnosed epileptic seizures during this period, the use of 2 independent databases enhances the likelihood of capturing all AVM-related hemorrhagic episodes. All patients in the SIPP-FIN cohort were present in the AVM database, and vice versa, within the overlapping time of these datasets (1987-2014).

### Definitions and Classifications

The trimesters were classified as up to h12 + 0 being the first, h12 + 1 to h28 + 0 the second, and from h28 + 1 until delivery the third. Puerperium was defined as the period from delivery to h12 (84 days) because of existing evidence regarding this period being associated with increased stroke risk.^14,15^ Fetal outcomes were evaluated regarding birth weight, Apgar points (1/5 minutes), umbilical cord arterial pH, need for intensive/intermediate care or resuscitation, and infant mortality. Maternal outcome was determined by the Glasgow Outcome scale at early follow-up (1-3 months after delivery) and modified Rankin scale last follow-up (latest follow-up data in medical records or in HRQOL questionnaire). Quantitative variables were handled continuous, except for AVM size, which was classified into small (0-1 inches), medium (1-2 inches), and large (>2 inches). AVM size was based on computed tomography angiography/digital subtraction angiography and was determined by the largest nidal diameter.

### Statistical Methods

Patients were categorized based on whether they had AVM rupture during pregnancy, delivery, or puerperium and were compared regarding categorical variables by using the Fisher exact test. For continuous variables either independent sample *t*-test or ANOVA with Bonferroni post hoc correction was used for the analyses of mean variance. Patients with missing variables were not included in the analyses of the variable in question. Statistical significance level was set at <0.05. Statistical analysis was performed using IBM SPSS Statistics for Windows (version 25, 64-bit edition).

### Ethical and Legal Considerations

The SIPP-FIN study is approved by the Ethics Committee of Helsinki University Hospital (HUS/2228/2016) and the register-keeping organizations the Finnish Institute of Health and Welfare (THL/750/7.05.00/2017) and Statistics Finland (TK-53-783-17, TK-53-591-20) granted permission to use register data. Helsinki University Hospital granted research permits to SIPP-FIN (HUS/163/2019) and the Helsinki AVM database (HUS3666/2017). Owing to Finnish legislation, pseudonymized raw data cannot be shared outside *blinded*. The study follows STROBE guidelines.

## RESULTS

### Main Characteristics

The study included 28 patients with symptomatic AVM, of whom 21 (75%) had AVM hemorrhage during pregnancy, delivery, or puerperium (Figure). Seven patients had AVM-related epileptic seizure. Mean follow-up time was 12.8 years (SD = 15.5). Mean age of patients with AVM hemorrhage during pregnancy, delivery, or puerperium (n = 21) was 27.8 years (SD = 5.4), and without hemorrhage (n = 7) 32.0 years (SD = 5.0), and for the whole cohort (n = 28) 28.9 years (SD = 5.5). Most patients were admitted between 1996 and 2016 (n = 13, 46.4%), 8 patients between 1976 and 1996 (28.6%), and 7 between 1950 and 1976 (25.0%). The anatomic characteristics of AVMs are illustrated in Table 1. Hemorrhages occurred mostly during the second (n = 9, 42.9% of all ruptures) or third trimester (n = 5, 23.8%). There were 2 ruptures during labor, 1 was an initial hemorrhage and the second a rebleed (first bleed during second trimester). Both were vaginal deliveries. One hemorrhage was related to an intranidal aneurysm (frontal ICH + intraventricular hematoma, h20 + 2). Four AVM ruptures (19.0%) occurred during first trimester and 2 during puerperium (9.5%). The most common presenting symptom for hemorrhage was severe headache (n = 13). Of the patients with no rupture during pregnancy, 5 (71.4% of the subcohort) were diagnosed because of epilepsy and 2 (28.6%) because of a focal neurologic symptom.

**FIGURE.**
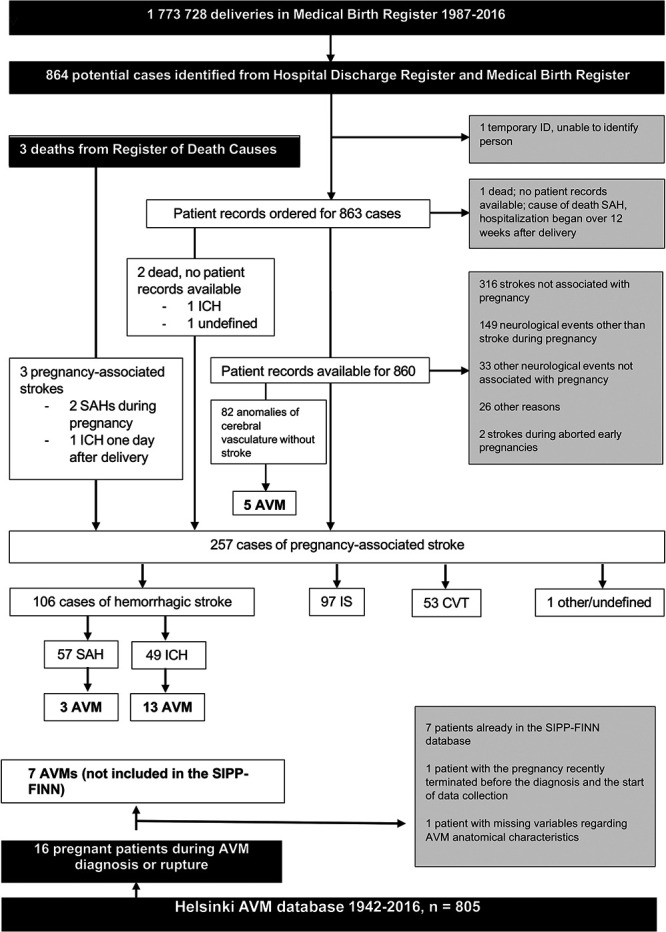
Flowchart of patient selection from the SIPP-FIN and the Helsinki AVM databases. AVM, arteriovenous malformation; CVT, cerebral venous thrombosis; ICH, intracerebral hemorrhage; IS, ischemic stroke; SAH, subarachnoid hemorrhage; SIPP-FIN, Stroke in Pregnancy and Puerperium in Finland.

**TABLE 1.** AVM Characteristics and Treatment

AVM rupture during pregnancy, delivery, or puerperium			Total (%)	Fisher exact test (2-sided) *P*, OR 95% CI
	Yes	No		
	21 (75)	7 (25)	28 (100)	
Spetzler-Ponce (%)				*P* = .52
A	10 (48)	3 (43)	13 (46)	
B	7 (33)	4 (57)	11 (39)	
C	4 (19)	0 (0)	4 (19)	
Supratentorial	20 (95)	6 (86)	26 (93)	*P* = .44, OR = 0.7-1.2
Infratentorial	1 (4.8)	1 (14)	2 (7.1)	
Size				*P* = .61
Small (<1 inch)	11 (52)	2 (29)	13 (46)	
Medium (1-2 inches)	5 (36)	5 (71)	10 (36)	
Large (>2 inches)	5 (18)	0 (0)	5 (18)	
Major sinus occlusion	0 (0)	2 (29%)		*P* = .056, OR = 0.4-1.1
Associated aneurysm	4 (19)	0 (0)		*P* = .55, OR = 0.99-1.5

AVM, arteriovenous malformation; OR, odds ratio.

### AVM Treatment and Outcomes

AVM treatment modalities during pregnancy are illustrated in Table 2. Twenty patients (71.4%) were microsurgically treated during the study period, 4 (14.3%) received radiotherapy (none of them during pregnancy), and 8 (28.6%) endovascular treatment. In 10 (35.7%) patients, the treatment took place either during pregnancy (n = 7, 25.0%) or puerperium (n = 3, 14.3%). All received microsurgical treatment at some point of their AVM treatment. There was no significant difference between functional outcome between mothers who were treated during pregnancy or afterwards; however, multivariate analyses could not be conducted because of limited sample size. There were relatively more Spetzler-Ponce B lesions in the conservatively managed group (n = 10) compared with the actively treated (n = 1), *P* = .041 (95% CI = 1.2-3.5).

**TABLE 2.** Descriptive Table About Patients Whose AVMs Were Treated During Pregnancy or Puerperium

Spetzler-Ponce	A	B	C
N (total)	7	1	2
Venous drainage			
Only superficial	5	1	1
Associated aneurysms	2	0	0
Bleeding during pregnancy	6	1	2
Epileptic seizures	2	0	0
Treatment modality during pregnancy (P if during puerperium)			
Embolization	0	0	2
Microsurgery	5	0	0
Embo + microsurgery	2P	1P	0
Outcome at last follow-up			
mRS 0-1	3	0	1
mRS 2-5	2	1	1
Dead (AVM-related)	1	0	0
Missing value	1	0	0
Trimester or puerperium during AVM treatment			
First	1	0	1
Second	0	0	0
Third	4	0	1
Puerperium	2	1	0

AVM, arteriovenous malformation; mRS, modified Rankin scale.

Three patients were treated conservatively within the follow-up period, 2 of them never had hemorrhage (diagnosis based on epileptic seizures) and 1 had several bleeding episodes during pregnancy in the 1960's but because of the eloquent location the lesion was not operated on. The maternal outcomes were better in the nonhemorrhagic group (Table 3). The 4 patients who had died during follow-up because of AVM-related reasons had been initially treated between 1960 and 1988. Three of them had a postoperative hydrocephalus. One had AVM hemorrhage altogether 8 times and was operated 5 times but died eventually due to a massive rebleed. Two patients in the nonhemorrhagic group later had AVM hemorrhage unrelatedly to pregnancy.

**TABLE 3.** Obstetric Management and Outcomes

AVM rupture during pregnancy/puerperium				
	Yes	No	Total	Independent sample *t* test/ANOVA (95% CI)
Mean age, y (SD)	27.8 (5.4)	32.0 (5.0)	28.9 (5.5)	*P* = .082 (−0.6 to 8.9)
Mean (SD)				
Previous childbirths, n	1.3 (1.6)	0.8 (0.8)	1.2 (1.4)	*P* = .50 (−1.7 to 1.8)
Previous pregnancies, n	1.56 (1.8)	1.6 (0.6)	1.6 (1.6)	*P* = .96 (−2.1 to 1.0)
Previous miscarriages, n	0.14 (0.4)	0.40 (0.6)	0.21 (0.4)	*P* = .25 (−1.6 to 2.3)
Previous abortions, n	0.14 (0.5)	0.40 (0.6)	0.21 (0.5)	*P* = .37 (−1.7 to 1.7)
First time pregnancy, n	5 (31)	0 (0)	5 (24)	*P* = .28 (−1.6 to 2.2)
Birth modality (n)				Fisher's exact test (2-sided) *P* = .46
Spontaneous vaginal	11 (52)	3 (43)	14 (50)	
Vacuum extraction	1 (4.8)	2 (29)	3 (11)	
Elective c-section	7 (33)	1 (14)	8 (29)	
Urgent c-section	1 (4.8)	1 (14)	2 (7.1)	
Emergency c-section	1 (4.8)	0 (0)	1 (3.6)	

Fetal outcomes				
	AVM rupture	No AVM rupture		ANOVA/*t* test
Mean (SD)				
Apgar 1 min	8.3 (2.0)	8.4 (1.5)	8.3 (1.9)	*P* = .91 (−2.0 to 2.2)
Apgar 5 min	8.1 (2.4)	8.6 (1.1)	8.2 (2.1)	*P* = .65 (−1.9 to 2.9)
Umbilical arterial pH	7.30 (0.08)	7.24 (0.17)	7.29 (0.01)	*P* = .45 (−1.7 to 2.8)

AVM, arteriovenous malformation.

### Obstetric Management and Outcomes

Only 5 (17.8%) mothers had not been previously pregnant. Most had either had 1 (n = 7) or 2 (n = 6) pregnancies before the index pregnancy. Delivery was on average during 38^th^ week (range h30 + 5 to h41 + 0); half were spontaneous vaginal births. One patient, diagnosed in the 1970s, had hemorrhage in early second trimester which led to death of the fetus and mother. This was the only case where the fetus died. Three newborns had Apgar <7 at 1 minutes, 2 were with mothers who had AVM hemorrhage at third trimester and 1 after full-term pregnancy during which mother had epileptic seizures. Three children required intermediate care during their perinatal period, 2 required assisted ventilation, and none required intensive care unit treatment. One child was born <2500 g of weight, after AVM rupture at h30 + 5 leading to emergency c-section, after which the AVM was operated on. The obstetric management and fetal outcomes are demonstrated in Tables 3 and 4. Regarding pregnancy complications, we had data on hyperglycemic disorders for 19 mothers: 5 (26%) underwent further diagnostics for gestational diabetes, and 2 (10%) had it diagnosed. Further data on pregnancy complications were available for 12 mothers: none had pre-eclampsia, eclampsia, HELLP, or other hypertensive disorders of pregnancy.

**TABLE 4.** Maternal Outcomes

AVM rupture during pregnancy			(%)	Fisher exact test, 2-sided *P*, 95% CI
	Yes	No	Total	
	21	7	28	
Maternal outcomes				
GOS at 2-4 mo				*P* = .52, OR = 0.6-1.9
Good recovery	13 (65)	5 (71)	18 (67)	
Moderate disability	1 (5.0)	1 (14)	2 (7.4)	
Severe disability	4 (20)	0 (0)	4 (15)	
Dead	2 (10)	1 (14)	3 (11)	
mRS at last follow-up				*P* = .33, OR = 0.3-2.0
No symptoms	5 (25)	3 (43)	8 (30)	
No disability despite symptoms	6 (30)	0 (0)	6 (22)	
Slight disability	1 (5.0)	1 (14)	2 (7.4)	
Moderate disability	3 (15)	0 (0)	3 (11)	
Moderately severe disability	1 (5.0)	0 (0)	1 (3.7)	
Dead	4 (20)	3 (43)	7 (26)	
AVM-related deaths	3 (14)	1 (14)	4 (14)	

AVM, arteriovenous malformation; GOS, Glasgow Outcome Scale; mRS, modified Rankin scale; OR, odds ratio.

### Patient Series From 1956 to 1989

Based on the search from our retrospective AVM database, we identified 11 patients who were admitted before 1990. Only 2 were treated during pregnancy between 1956 and 1989 (Table 5). Eight mothers during this period gave spontaneous vaginal births, and there was only 1 pregnancy termination. Despite mostly conservative management in both neurosurgical and obstetric fields, most mothers (81%) had favorable functional outcomes (Glasgow Outcome scale 1-2) at 3-month follow-up from the delivery (Table 5). Two mothers had died because of the AVM, 1 because of venous infarction after AVM operation during puerperium; the second despite operative and ventriculostomy treatment after severe initial AVM hemorrhage and hydrocephalus.

**TABLE 5.** The Symptomatic AVM Patients Treated Before 1990

Patient	Age	Admission	Trimester^a^	Symptom	Management during pregnancy (AVM/OBGYN)	GOS 3 mo^b^
1	37	1989	II	B, SE	CON/SPONT h41	1
2	33	1989	II	SE	CON/Elective c-section h38	1
3^c^	28	1988	III	SE	CON/SPONT F-T	5
4	24	1979	I	B	Oper/Termination h14	5
5	23	1973	I	B	Oper/SPONT F-T	1
6	35	1970	III	B	CON/SPONT h38	1
7	28	1966	Labor	B	CON/SPONT	1
8	34	1966	II	SE	CON/SPONT	1
9	17	1966	II	B	CON/SPONT F-T	2
10	30	1960	I	B	CON/SPONT	1
11	22	1956	III	B	CON/Elective c-section	1

AVM, arteriovenous malformation; B, bleeding; CON, conservative; F-T, full-term (h39-h40 + 6); GOS, Glasgow Outcome Scale; OBGYN, obstetric management; SE, symptomatic epilepsy; SPONT, spontaneous vaginal birth.

a Time when AVM became symptomatic.

b Glasgow Outcome Scale at 3 months after delivery.

c The patient was operated during puerperium after first successfully giving birth spontaneously. She developed sinus occlusion after the operation and died because of venous infarction.

## DISCUSSION

Based on our findings most pregnancy-related AVM ruptures happened during the last two-thirds of pregnancy, and most mothers had already had previous pregnancies. Outcomes of both mothers and newborns were similar between the hemorrhage and epileptic seizure cohorts. The last AVM-related fatality during pregnancy, delivery, or puerperium was in 1988. Despite the older patient series illustrating mostly conservative management in both the neurosurgical and obstetric fields, most mothers had favorable outcomes at early follow-up.

### Relationship Between Previous Pregnancy, Trimester, and AVM Rupture

Most of the AVM ruptures happened during the second and third trimester. Previous literature reports accumulation of hemorrhages toward the end of pregnancy.^3,8,16-18^ The finding is interesting because if there was no connection between pregnancy and AVM rupture, supposedly, hemorrhages should happen unrelatedly to the trimester. Furthermore, most of the mothers (76%) had had previous pregnancies before the index pregnancy. The possible explanatory factors during pregnancy on the increased rupture risk toward the second and third trimester could include the increased maternal blood volume, venous blood pressure and cardiac output, and increase in estrogen levels.^19-22^ However, pregnancy-related AVM hemorrhage is a rare event, and insufficient amount of patient-level data exist. This has made AVM rupture risk calculations difficult because optimally they should consider the pre-existing risk factors^23^; and this requires sufficient sample size and data. Overall, the age of the mothers was in line with the maternity trend in Finland, in which the age of mothers has increased and the fertility rate declined.^24^ Supposedly, if pregnancy was associated with increased rupture risk, we should see a trend toward decreasing numbers of pregnancy-related hemorrhages. However, given the possible risk of missed diagnoses in the earliest decades of our study, it is possible that this phenomenon cannot be illustrated by our data. Combining existing patient series between neurosurgical units could merit a large enough sample to facilitate these estimations.

### Outcomes and Treatment Strategy

Survival of preterm infants improves steadily with gestational age and also prognosis has steadily improved during our study period owing to advances in neonatal care.^25,26^ Balancing between fetal development and the timing of AVM treatment needs to be evaluated individually. In our series, there was only one case where the child died because of AVM hemorrhage during first trimester and pregnancy termination one month after. The case was treated in the 1970s, after which neonatal care has developed significantly; however, even with modern intensive care unit capabilities, preterm fetuses < h23 cannot be saved. We witnessed 1 case with rerupture, which demonstrated that conservative management of once bled AVM can be detrimental because previous bleeding is a well-known risk factor for future ruptures.^27^ Being closer to the window of neonatal care could enable a waiting time of a couple of weeks; however, several months might increase the rerupture risk over an acceptable limit. Considering the whole study period, the rate of c-sections has increased in Finland; however, lately the overall rate of vaginal deliveries after c-section has been increasing.^28^ Currently, the rate of c-sections in Finland is around 20%.^28^ Notably, despite most mothers before the 1990s giving spontaneous vaginal births, the outcomes were mostly favorable and the change in the birth modalities more likely illustrates the developments in the obstetric and neonatal fields rather than change in the obstetric recommendations for AVM mothers. The prevailing obstetric guidelines in our institute regarding delivery methods for patients with unruptured AVMs give the decision-making autonomy to the patient.^29^ Yet, the rerupture during the vaginal delivery indicates that despite favorable outcomes, spontaneous vaginal birth after AVM rupture can be problematic. Changes to current recommendations cannot be made based only on this patient series, and multidisciplinary approach to treatment decisions is recommendable.^30^ Regarding unruptured AVMs, future studies are needed to clarify the AVM rupture risk related to delivery method.

The Canadian stroke Best Practice guideline recommends treating symptomatic low-grade AVMs during pregnancy in a same time frame as in nonpregnant women.^30^ For ruptured AVMs, they recommend treating the mother in the best available modality, regardless of pregnancy status. It is noted, however, that if the gestational age of the fetus is viable regarding favorable outcomes, a multidisciplinary discussion should be considered if the AVM treatment requires methods that compromise the fetus. These potential risks include radiation and contrast exposure and potential blood loss during surgery.^30^ Notably, pregnancy is no longer considered a contraindication for endovascular procedures^30^; however, potential radiation-induced side effects after AVM embolization remain unclear.^31^ In our series, the fetuses whose mothers had had endovascular treatment during pregnancy were born healthy, but we lack long-term outcome data.

### Parity and Lifestyle Associated Risk Factors

Most mothers had had a previous pregnancy. Parity in a dose-response relationship has been associated with increased risk of ICH and subarachnoid hemorrhage.^32^ The underlying reasons are not well understood. Current theories hypothesize that changes in physical and environmental factors during and after pregnancy and delivery could be one reason.^32,33^ Additionally to the possible effects of pregnancy-induced hypertension and decreased vascular resistance,^34,35^ the new demands of family life often with physical and psychological strain can increase the risk of cardiovascular disease.^36,37^ This effect is apparent also with the spouses, which supports the environmental theory of the effects of multiparity. Notably, our cohort represents women from different decades, during which there have been major changes in lifestyle. Medical treatment and primary prevention have improved, simultaneously the birth rate has steadily decreased, and the age of first-time mothers increased. There are significant differences between the age of first-time mothers between socioeconomical classes and decades. We did not analyze socioeconomic data; therefore, strong interpretations about multiparity in our patient cohort cannot be made.

### Limitations

We had no information on patients with asymptomatic AVMs and thus were limited to analyze only patients with symptomatic AVMs. In the modern era of neuroimaging with increasing number of incidental AVMs, these patients can possibly be explored in the future. Limited number of patients posed challenges for statistical analyses. Owing to small subgroup sizes, we refrained from conducting multivariate analyses. We optimized the accuracy of our data by excluding patients with missing data. Some patients were treated before 1990s, yet exploring the differences in patient characteristics, treatment, and outcomes by treatment decade showed no significant differences in maternal outcomes. Notably, advancements in neurosurgical, obstetric, and neonatal care over the study period may influence outcomes. Modern patient cohorts could possibly experience more favorable results. Still, including the older patient cohort helped understand different treatment strategies because most patients in these years were treated conservatively and had spontaneous vaginal births. Most patients were imaged with computed tomography after 1978, before which the diagnosis was based on carotid angiography and/or blood in cerebrospinal fluid; thus, this marks an important turning point in the diagnostics. Before the developments in neuroimaging, some pregnancy-related AVM hemorrhages and AVM-related epilepsies have possibly remained undiagnosed. More data from other institutions are crucial for the generalizability of our results. Furthermore, combining data across institutes could merit large enough sample size to conduct multivariate analyses.

## CONCLUSION

In the cases of progressing neurologic symptoms or declining condition of the mother after AVM rupture during pregnancy, active treatment is needed. If mother's condition is stable or the AVM is diagnosed unruptured, patient and doctors are faced with a unique situation, which requires balancing between the risks of conservative management and active treatment. Most mothers treated during pregnancy in our patient cohort had had an AVM hemorrhage, and despite this, most of them and their newborn babies recovered well. There was no significant difference between active and observational AVM treatment during pregnancy regarding the maternal or fetal outcomes. We reported 1 initial and 1 rebleed during vaginal delivery. An increased number of multiparas and the association of rupture toward the end of pregnancy could indicate a relationship with multiparity and increased rupture risk.

## COMMENTS

Among many aspects of brain arteriovenous malformation (AVM), the incidence of AVM rupture and its impact on the mother and the fetus or newborn during pregnancy and delivery have been the least known topics. This retrospective study focusing on brain AVM during pregnancy and puerperium was based on the National Hospital Discharge Register and Medical Birth Register in Finland from 1987 to 2016, with an additional AVM registry between 1942 and 2014. The study provides crucial insights into symptomatic AVM, particularly the occurrence of AVM rupture associated with pregnancy, the outcomes of mothers along with their fetus/newborns following AVM rupture, and the indicated interventions for AVM during this period. In essence, the study found the majority of pregnant women with AVM rupture had at least one previous pregnancy without a hemorrhage event. More than 2/3 of AVM ruptures occurred during the second and third trimester and 9.5% during the vaginal delivery. Delivery on average was during gestation of the 38th week, a mature newborn. There was no significant outcome difference between the mothers who underwent AVM therapeutic procedures and those who received conservative management. Most of the newborns had good outcomes. Despite the limited number of symptomatic AVM cases in this study, its findings will impact the neurosurgical and obstetric management of pregnancy in women with AVM.

**Peng Roc Chen**

*Houston, Texas, USA*

## References

[1] Dias MS Sekhar LN. Intracranial hemorrhage from aneurysms and arteriovenous malformations during pregnancy and the puerperium. Neurosurgery. 1990;27(6):855-866; discussion 865-866. 2274125 10.1097/00006123-199012000-00001

[2] Khan M Wasay M. Haemorrhagic strokes in pregnancy and puerperium. Int J Stroke. 2013;8(4):265-272. 22863273 10.1111/j.1747-4949.2012.00853.x

[3] Porras JL Yang W Philadelphia E Hemorrhage risk of brain arteriovenous malformations during pregnancy and puerperium in a North American cohort. Stroke. 2017;48(6):1507-1513. 28487334 10.1161/STROKEAHA.117.016828

[4] Kittner SJ Stern BJ Feeser BR Pregnancy and the risk of stroke. N Engl J Med. 1996;335(11):768-774. 8703181 10.1056/NEJM199609123351102 PMC1479545

[5] Vest T Rantanen K Verho L Etiology of intracerebral hemorrhage during pregnancy or puerperium: a nationwide study. Eur J Neurol. 2024;31(3):e16012. 37532682 10.1111/ene.16012 PMC11235636

[6] Zhu D Zhao P Lv N Rupture risk of cerebral arteriovenous malformations during pregnancy and puerperium: a single-center experience and pooled data analysis. World Neurosurg. 2018;111:e308-e315. 29258932 10.1016/j.wneu.2017.12.056

[7] Tiel Groenestege AT Rinkel GJ van der Bom JG Algra A Klijn CJ. The risk of aneurysmal subarachnoid hemorrhage during pregnancy, delivery, and the puerperium in the Utrecht population: case-crossover study and standardized incidence ratio estimation. Stroke. 2009;40(4):1148-1151. 19211489 10.1161/STROKEAHA.108.539700

[8] Horton JC Chambers WA Lyons SL Adams RD Kjellberg RN. Pregnancy and the risk of hemorrhage from cerebral arteriovenous malformations. Neurosurgery. 1990;27(6):867-872; discussion 871-872. 2274126 10.1097/00006123-199012000-00002

[9] Karlsson B Johansson AV Jokura H Risk for brain arteriovenous malformation rupture during pregnancy and puerperium. Neurosurgery. 2023;93(4):918-923. 37074063 10.1227/neu.0000000000002496

[10] De Maria L Serioli S Fontanella MM. Brain arteriovenous malformations and pregnancy: a systematic review of the literature. World Neurosurg. 2023;177:100-108. 37355173 10.1016/j.wneu.2023.06.065

[11] Karjalainen L Tikkanen M Rantanen K Stroke in pregnancy and puerperium: validated incidence trends with risk factor analysis in Finland 1987-2016. Neurology. 2021;96(21):e2564-e2575. 33827961 10.1212/WNL.0000000000011990

[12] Korhonen A Verho L Aarnio K Subarachnoid hemorrhage during pregnancy and puerperium: a population-based study. Stroke. 2023;54(1):198-207. 36321452 10.1161/STROKEAHA.122.039235

[13] Spetzler RF Martin NA. A proposed grading system for arteriovenous malformations. J Neurosurg. 1986;65(4):476-483. 3760956 10.3171/jns.1986.65.4.0476

[14] Kamel H Navi BB Sriram N Hovsepian DA Devereux RB Elkind MS. Risk of a thrombotic event after the 6-week postpartum period. N Engl J Med. 2014;370(14):1307-1315. 24524551 10.1056/NEJMoa1311485 PMC4035479

[15] Tang CH Wu CS Lee TH Preeclampsia-eclampsia and the risk of stroke among peripartum in Taiwan. Stroke. 2009;40(4):1162-1168. 19228854 10.1161/STROKEAHA.108.540880

[16] Forster DM Kunkler IH Hartland P. Risk of cerebral bleeding from arteriovenous malformations in pregnancy: the Sheffield experience. Stereotact Funct Neurosurg. 1993;61(Suppl 1):20-22. 8115751 10.1159/000100655

[17] Gross BA Du R. Hemorrhage from arteriovenous malformations during pregnancy. Neurosurgery. 2012;71(2):349-356; discussion 355-356. 22472554 10.1227/NEU.0b013e318256c34b

[18] Liu XJ Wang S Zhao YL Risk of cerebral arteriovenous malformation rupture during pregnancy and puerperium. Neurology. 2014;82(20):1798-1803. 24759847 10.1212/WNL.0000000000000436 PMC4035708

[19] Helms AK Kittner SJ. Pregnancy and stroke. CNS Spectr. 2005;10(7):580-587. 16155514 10.1017/s1092852900010221

[20] Nevo O Soustiel JF Thaler I. Cerebral blood flow is increased during controlled ovarian stimulation. Am J Physiol Heart Circ Physiol. 2007;293(6):H3265-H3269. 17965286 10.1152/ajpheart.00633.2007

[21] Longstreth WT Nelson LM Koepsell TD van Belle G. Subarachnoid hemorrhage and hormonal factors in women. A population-based case-control study. Ann Intern Med. 1994;121(3):168-173. 8017743 10.7326/0003-4819-121-3-199408010-00002

[22] Meah VL Cockcroft JR Backx K Shave R Stohr EJ. Cardiac output and related haemodynamics during pregnancy: a series of meta-analyses. Heart. 2016;102(7):518-526. 26794234 10.1136/heartjnl-2015-308476

[23] Chen Y Han H Meng X Development and validation of a scoring system for hemorrhage risk in brain arteriovenous malformations. JAMA Netw Open. 2023;6(3):e231070. 36857052 10.1001/jamanetworkopen.2023.1070 PMC9978947

[24] Roustaei Z Raisanen S Gissler M Heinonen S. Fertility rates and the postponement of first births: a descriptive study with Finnish population data. BMJ Open. 2019;9(1):e026336. 10.1136/bmjopen-2018-026336 PMC6340426 30782758

[25] Draper ES Manktelow B Field DJ James D. Prediction of survival for preterm births by weight and gestational age: retrospective population based study. BMJ. 1999;319(7217):1093-1097. 10531097 10.1136/bmj.319.7217.1093 PMC28258

[26] Whitburn RH Laishley RS Jewkes DA. Anaesthesia for simultaneous caesarean section and clipping of intracerebral aneurysm. Br J Anaesth. 1990;64(5):642-645. 2354104 10.1093/bja/64.5.642

[27] Hernesniemi JA Dashti R Juvela S Vaart K Niemela M Laakso A. Natural history of brain arteriovenous malformations: a long-term follow-up study of risk of hemorrhage in 238 patients. Neurosurgery. 2008;63(5):823-831; discussion 829-831. 19005371 10.1227/01.NEU.0000330401.82582.5E

[28] Vaajala M Liukkonen R Ponkilainen V Kekki M Mattila VM Kuitunen I. The rates of vaginal births after cesarean section have increased during the last decades: a nationwide register-based cohort study in Finland. Arch Gynecol Obstet. 2023;308(1):157-162. 37016061 10.1007/s00404-023-07010-y PMC10192182

[29] Davidoff CL Lo Presti A Rogers JM Risk of first hemorrhage of brain arteriovenous malformations during pregnancy: a systematic review of the literature. Neurosurgery. 2019;85(5):e806-e814. 31149721 10.1093/neuros/nyz175

[30] Ladhani NNN Swartz RH Foley N Canadian stroke best practice consensus statement: acute stroke management during pregnancy. Int J Stroke. 2018;13(7):743-758. 30021491 10.1177/1747493018786617

[31] Yan KL Ko NU Hetts SW Maternal and fetal outcomes in women with brain arteriovenous malformation rupture during pregnancy. Cerebrovasc Dis. 2021;50(3):296-302. 33640891 10.1159/000513573 PMC8159887

[32] Jung SY Bae HJ Park BJ Yoon BW., Acute Brain Bleeding Analysis Study Group. Parity and risk of hemorrhagic strokes. Neurology. 2010;74(18):1424-1429. 20335561 10.1212/WNL.0b013e3181dc13a5

[33] Grundy E Tomassini C. Fertility history and health in later life: a record linkage study in England and Wales. Soc Sci Med. 2005;61(1):217-228. 15847974 10.1016/j.socscimed.2004.11.046

[34] Castelao JE Gago-Dominguez M. Risk factors for cardiovascular disease in women: relationship to lipid peroxidation and oxidative stress. Med Hypotheses. 2008;71(1):39-44. 18308480 10.1016/j.mehy.2007.10.016

[35] Juntunen K Kirkinen P Kauppila A. The clinical outcome in pregnancies of grand grand multiparous women. Acta Obstet Gynecol Scand. 1997;76(8):755-759. 9348253 10.3109/00016349709024342

[36] Hardy R Lawlor DA Black S Wadsworth ME Kuh D. Number of children and coronary heart disease risk factors in men and women from a British birth cohort. BJOG. 2007;114(6):721-730. 17516964 10.1111/j.1471-0528.2007.01324.x

[37] Ness RB Harris T Cobb J Number of pregnancies and the subsequent risk of cardiovascular disease. N Engl J Med. 1993;328(21):1528-1533. 8267704 10.1056/NEJM199305273282104

